# Primary Amebic Meningoencephalitis Mimicking Acute Bacterial Meningitis: Survival Following Early Empirical Therapy

**DOI:** 10.7759/cureus.106656

**Published:** 2026-04-08

**Authors:** Soukaina Mekhchoun, Yasmina Zakaria, Khaoula Balili, Mohamed Chraa, Nissrine Louhab

**Affiliations:** 1 Department of Neurology, Mohammed VI University Hospital, Faculty of Medicine and Pharmacy, Cadi Ayyad University, Marrakech, MAR

**Keywords:** acute necrotizing meningoencephalitis, early empirical treatment, naegleria fowleri, primary amebic meningoencephalitis, survival

## Abstract

Primary amebic meningoencephalitis (PAM) is a rare but rapidly fatal infection of the central nervous system caused by the thermophilic free-living amoeba *Naegleria fowleri*. The disease often presents with clinical and cerebrospinal fluid (CSF) findings that closely mimic acute bacterial meningitis, making early diagnosis particularly challenging.

We report the case of a 29-year-old woman with poorly controlled type 1 diabetes who presented with acute fever, severe headache, vomiting, and meningeal signs. Within 24 hours, her condition progressed to visual and auditory hallucinations, generalized tonic-clonic seizures, and decreased consciousness. CSF analysis revealed neutrophilic pleocytosis, elevated protein levels, and low glucose concentration, initially suggesting bacterial meningitis. Brain MRI demonstrated bilateral cortical-subcortical hyperintense lesions involving the frontal, insular, and temporal regions, with associated microhemorrhages, consistent with necrotizing meningoencephalitis. Further history revealed a recent nasal ablution using warm lake water. Microscopic examination of CSF showed findings suggestive of an amoeboid trophozoite, raising suspicion for *N. fowleri* infection, although definitive parasitological confirmation could not be obtained. Empirical therapy with liposomal amphotericin B, fluconazole, and rifampicin was initiated promptly. The patient showed progressive clinical improvement, with complete recovery of consciousness and marked radiological resolution of lesions on follow-up imaging.

This case highlights the diagnostic challenges posed by PAM and underscores the importance of early clinical suspicion and prompt empirical therapy in suspected cases, even in the absence of definitive laboratory confirmation.

## Introduction

Primary amebic meningoencephalitis (PAM) is a rare but rapidly fatal infection of the central nervous system caused by the thermophilic free-living amoeba *Naegleria fowleri *[1]. The organism is commonly found in warm freshwater environments and infects humans when contaminated water enters the nasal cavity, allowing the amoeba to invade the olfactory neuroepithelium and migrate through the cribriform plate into the brain [1,2]. In U.S. epidemiologic series, PAM remains exceptionally uncommon, with fewer than 10 reported cases annually, and predominantly affects children and adolescents with a marked male predominance [3,4].

Although PAM is uncommon, it is associated with an extremely high mortality rate exceeding 95% and a rapidly progressive clinical course that often leads to death within a few days after symptom onset [3,4]. The disease typically presents with symptoms resembling acute bacterial meningitis, including fever, headache, vomiting, seizures, and altered mental status, which frequently leads to delayed recognition and misdiagnosis in the early stages [1,5].

Definitive diagnosis generally relies on the identification of trophozoites in cerebrospinal fluid (CSF) or the detection of the organism using molecular techniques such as polymerase chain reaction (PCR) assays [5]. However, these diagnostic tools may not always be readily available, particularly in the early stages of the disease, and a lack of access to confirmatory testing in many settings can make diagnosis especially difficult [6]. Given the fulminant progression of PAM, early clinical suspicion and prompt initiation of empirical therapy may represent one of the few opportunities to improve survival in this otherwise frequently fatal infection [4,5].

In this report, we describe a presumptive case of PAM mimicking acute bacterial meningitis, with a favorable clinical course following early empirical therapy despite the absence of definitive parasitological or molecular confirmation.

## Case presentation

A 29-year-old woman with poorly controlled type 1 diabetes mellitus presented to the emergency department with an acute headache, fever (39.4°C), and vomiting for 12 hours. Neurological examination revealed meningeal signs (Brudzinski and Kernig) and anosmia, suggestive of olfactory nerve (cranial nerve I) involvement. Within 24 hours, the patient developed visual and auditory hallucinations, followed by a generalized tonic-clonic seizure and a decrease in the Glasgow Coma Scale (GCS) score to 9. Further history revealed that the patient had recently performed nasal ablutions using warm lake water as part of a religious ritual. This exposure, with direct nasal irrigation, constituted a recognized route of infection for *N. fowleri*.

**Figure 1:**
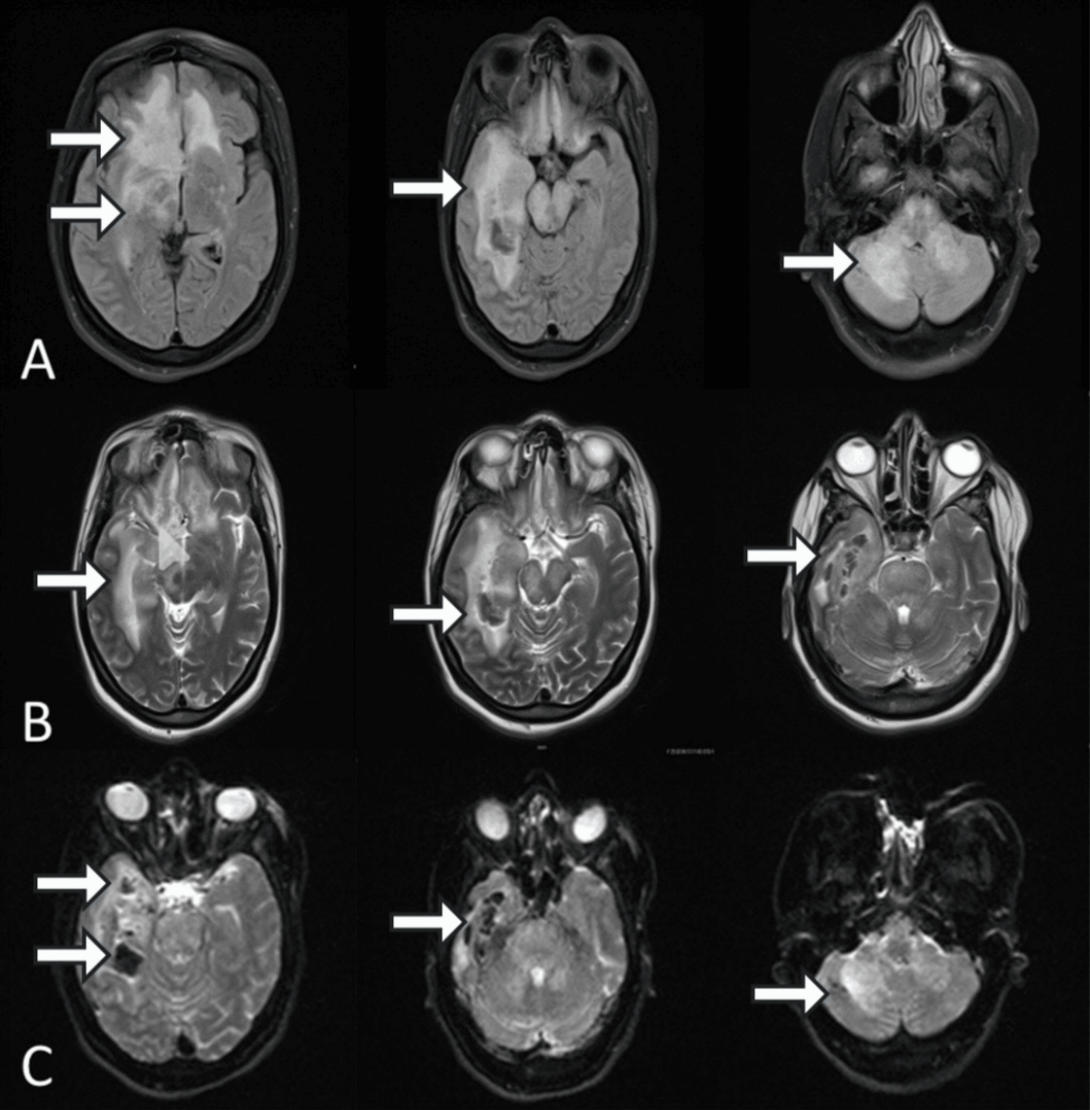
Brain MRI at admission (A) Axial FLAIR-weighted images demonstrate multiple bilateral cortical-subcortical hyperintense lesions predominantly involving the frontal lobes and insular cortices, with extension to the temporal lobes, associated with surrounding vasogenic edema (white arrows). (B) Axial T2-weighted images confirm extensive hyperintense lesions with heterogeneous signal intensity, suggesting areas of necrosis in the frontal, insular, and temporal regions (white arrows). (C) Susceptibility-weighted imaging (SWI)/T2-weighted images reveal multiple punctate hypointense foci within the lesions, consistent with microhemorrhages (white arrows).

Brain magnetic resonance imaging (MRI) performed at admission demonstrated multiple bilateral cortical-subcortical hyperintense lesions on fluid-attenuated inversion recovery (FLAIR) and T2-weighted sequences, involving the frontal lobes, insular cortices, and temporal lobes. These lesions were associated with surrounding vasogenic edema. Susceptibility-weighted imaging (SWI) and T2-weighted sequences revealed multiple punctate hypointense foci consistent with microhemorrhages. This radiological pattern, characterized by multifocal necrotic-hemorrhagic lesions in the bilateral frontal and insular regions, was compatible with acute necrotizing meningoencephalitis (Figure 1).

The CSF was clear in appearance. CSF analysis revealed a leukocyte count of 360 cells/mm^3^ with a predominance of neutrophils (90%) and 86 erythrocytes/mm^3^. CSF protein concentration was markedly elevated at 3.68 g/L, while glucose was reduced to 0.49 g/L compared with a simultaneous blood glucose level of 1.8 g/L. This inflammatory profile, characterized by neutrophilic pleocytosis with elevated protein and low glucose levels, mimicked acute bacterial meningitis. Laboratory findings are summarized in Table 1.

**Table 1:** Cerebrospinal fluid (CSF) analysis at admission

Parameter	Patient value	Reference range
CSF appearance	Clear	Normally clear
CSF white blood cells (cells/mm^3^)	360	0-5
CSF erythrocytes (cells/mm^3^)	86	0
CSF mononuclear cells (%)	10	60-100
CSF neutrophils (%)	90	0-6
CSF protein (g/L)	3.68	0.15-0.45
CSF glucose (g/L)	0.49	0.45-0.80
Blood glucose (g/L)	1.80	0.70-1.10
CSF/blood glucose ratio	0.27	>0.60

Initial Gram stain and bacterial cultures were negative. Viral PCRs for herpes simplex virus types 1 and 2 (HSV-1/2), enterovirus, cytomegalovirus (CMV), and varicella-zoster virus (VZV), as well as tuberculosis testing (GeneXpert and acid-fast bacilli (AFB) stain), were all negative. Fresh wet-mount microscopy showed clusters of inflammatory cells with granular cytoplasm and irregular contours, one of which exhibited a pseudopodial extension, raising suspicion of a free-living amoeboid organism. Although definitive morphological identification of a trophozoite was not possible, these findings were considered suggestive by the parasitology team and contributed to a presumptive rather than confirmed diagnosis.

Given the clinical deterioration, neuroimaging findings, and suggestive parasitological evidence, a presumptive diagnosis of PAM was established. Consequently, treatment was initiated on hospital day 2, prior to microbiological confirmation. The regimen included liposomal amphotericin B (3 mg/kg/day, intravenously), fluconazole (400 mg/day, intravenously), and rifampicin (400 mg/day, orally).

Clinically, the patient exhibited progressive improvement following treatment: she became afebrile by day 4, seizures ceased by day 7 under antiepileptic therapy, and full recovery of consciousness was achieved by day 10. She was discharged after 36 days without neurological deficits, except for persistent anosmia.

**Figure 2:**
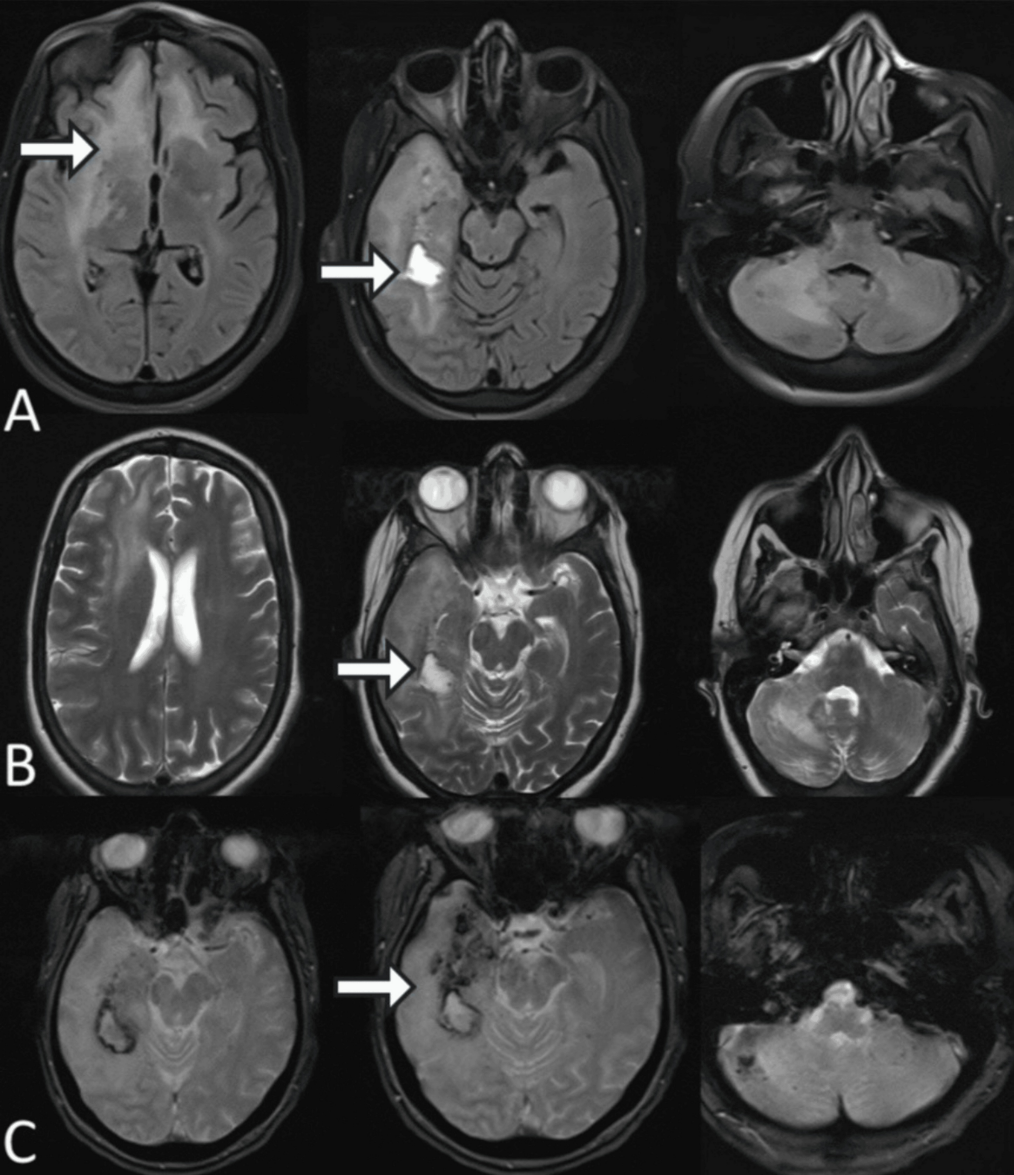
Follow-up brain MRI on day 15 showing marked radiological improvement (A) Axial FLAIR-weighted images demonstrate a marked reduction in the extent and signal intensity of previously described bilateral cortical-subcortical lesions involving the frontal, insular, and temporal regions, with significant regression of surrounding vasogenic edema (white arrows). (B) Axial T2-weighted images confirm interval improvement with decreased lesion size and reduced mass effect, with residual signal abnormalities in the temporal region (white arrows). (C) Susceptibility-weighted imaging (SWI)/T2-weighted images show a reduction in the number and extent of previously observed punctate hypointense foci, consistent with resolving microhemorrhages (white arrows).

Follow-up MRI performed on day 15 demonstrated a marked reduction in both the extent and signal intensity of the bilateral cortical-subcortical lesions involving the frontal lobes, insular cortices, and temporal lobes. Perilesional edema had significantly decreased, and SWI/T2 sequences revealed a reduction in microhemorrhages (Figure 2).

**Figure 3:**
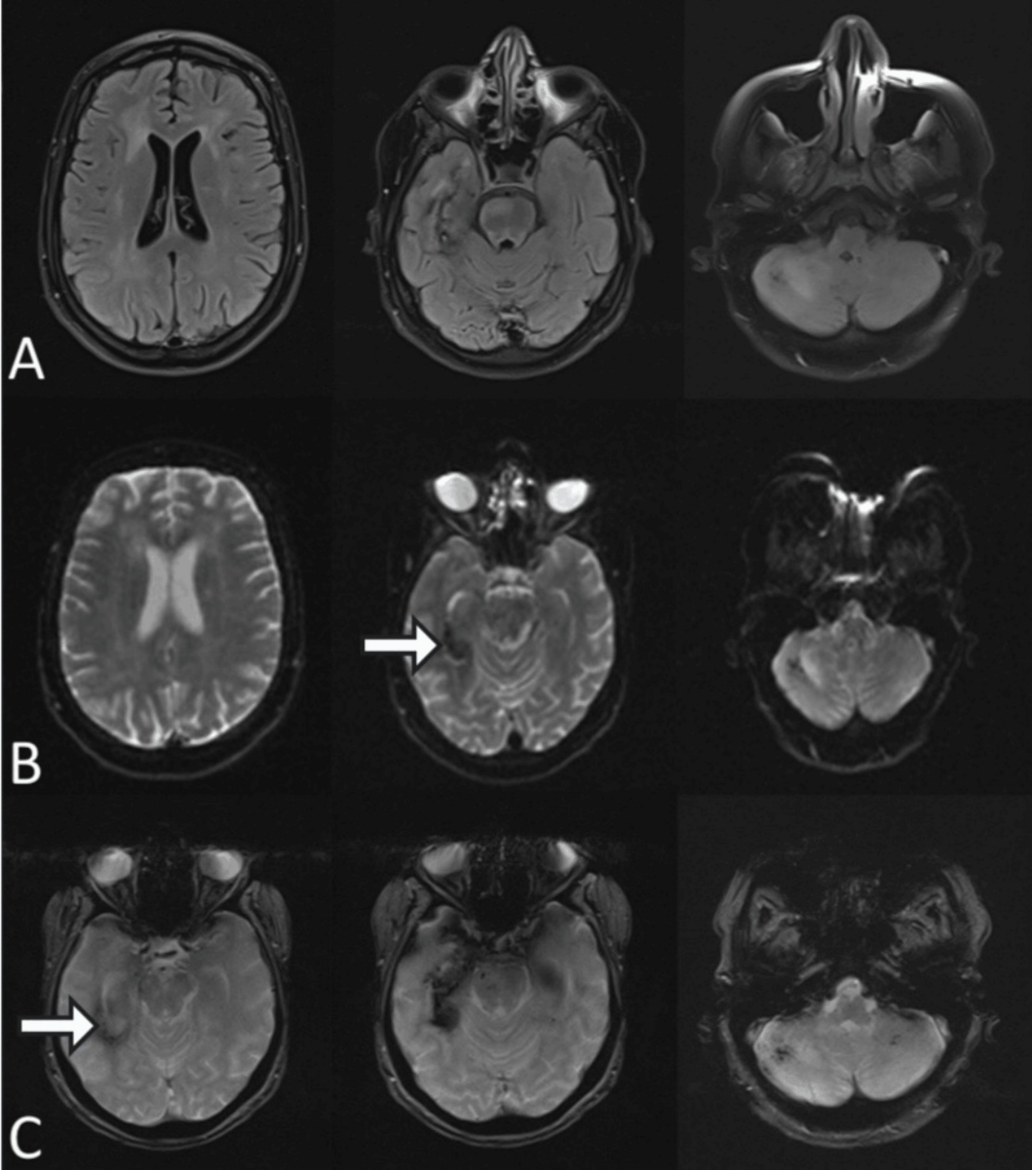
Follow-up brain MRI on day 35 showing near-complete resolution with minimal residual abnormalities (A) Axial FLAIR-weighted images show near-complete resolution of previously described bilateral cortical-subcortical hyperintense lesions, with normalization of signal intensity and resolution of surrounding vasogenic edema. (B) Axial T2-weighted images confirm marked radiological improvement with minimal residual signal abnormalities and no significant mass effect (white arrow). (C) Susceptibility-weighted imaging (SWI)/T2-weighted images demonstrate a marked reduction in previously observed hypointense foci, with minimal residual microhemorrhages (white arrow).

By day 35, MRI showed near-complete resolution of these lesions, with disappearance of edema and marked reduction to near-complete resolution of microhemorrhages (Figure 3).

## Discussion

PAM is a rare but highly lethal infection of the central nervous system caused by the thermophilic free-living amoeba *N. fowleri* [1]. The organism is widely distributed in warm freshwater environments and typically infects humans when contaminated water enters the nasal cavity, allowing the amoeba to penetrate the olfactory neuroepithelium and migrate through the cribriform plate into the brain [1,7,8].

The presence of anosmia in our patient may also be explained by this well-described route of invasion of *N. fowleri *through the olfactory neuroepithelium and olfactory nerves, which provide a direct pathway for the organism to reach the central nervous system [7,8]. This clinical finding may reflect involvement of the olfactory pathway during the early stages of infection.

Although PAM remains uncommon, it is associated with an extremely high mortality rate exceeding 95%, with most patients dying within a few days after symptom onset [3,4]. The rarity of the disease, combined with its rapidly progressive course, makes early diagnosis particularly challenging.

One of the main diagnostic difficulties is that the initial clinical presentation closely resembles acute bacterial meningitis. Patients typically present with fever, headache, vomiting, seizures, and meningeal signs, often accompanied by CSF findings such as neutrophilic pleocytosis, elevated protein levels, and decreased glucose concentrations [1,9]. In our case, the CSF profile was highly suggestive of bacterial meningitis, with marked neutrophilic pleocytosis, elevated protein levels, and low CSF glucose, which illustrates how easily PAM can be misdiagnosed in its early stages. In addition, trophozoites may be difficult to detect in CSF, particularly in the early stages of the disease or when microscopic examination is not immediately performed, which may further complicate the diagnostic process [5].

Neuroimaging findings in PAM are variable but may include cerebral edema, cortical abnormalities, and hemorrhagic lesions. MRI can demonstrate multifocal hyperintense lesions on T2-weighted and FLAIR sequences, sometimes associated with hemorrhagic changes reflecting the necrotizing inflammatory process caused by the organism [10]. In our patient, brain MRI revealed bilateral cortical-subcortical hyperintense lesions involving the frontal lobes, insular cortices, and temporal lobes, with associated microhemorrhages on SWI. This radiological pattern was consistent with necrotizing meningoencephalitis and supported the suspicion of PAM.

Definitive diagnosis of PAM is usually established by identifying trophozoites in CSF or by detecting the pathogen using molecular techniques such as PCR assays [11]. However, these diagnostic methods are not always available and may require specialized laboratory facilities, which can delay confirmation [6]. In our case, although microscopic examination revealed findings suggestive of an amoeboid trophozoite, definitive parasitological confirmation could not be obtained. The overall epidemiological, clinical, CSF, parasitological, and neuroimaging findings were highly suggestive of PAM, which remained the most likely diagnosis in this case. In rapidly progressive meningoencephalitis with compatible epidemiological exposure, characteristic CSF findings, and suggestive neuroimaging abnormalities, a presumptive diagnosis of PAM may be considered in the appropriate clinical context, even in the absence of definitive laboratory confirmation [5].

Because PAM progresses rapidly and carries a very high mortality rate, several authors emphasize the importance of starting empirical therapy as soon as the disease is strongly suspected. Several reported survivors of PAM received early amphotericin B-based combination therapy, including regimens containing fluconazole, rifampicin, or miltefosine [2,12,13]. These observations suggest that treatment should not be delayed when clinical suspicion for PAM is high, even in the absence of definitive laboratory confirmation.

Our patient received early empirical therapy with liposomal amphotericin B, fluconazole, and rifampicin based on the clinical, radiological, and epidemiological findings suggestive of PAM. The favorable clinical evolution observed in this case, with progressive neurological recovery and near-complete radiological resolution, supports the importance of early recognition and prompt initiation of treatment.

This case also illustrates the characteristic clinical pattern often described in PAM, including recent nasal exposure to lake water, neutrophilic CSF mimicking bacterial meningitis, and rapidly progressive neurological deterioration.

Similar diagnostic challenges have been described in previously reported cases, highlighting the need for clinicians to maintain a high index of suspicion when confronted with rapidly progressive meningoencephalitis, particularly in patients with a history of recent nasal exposure to contaminated freshwater, such as lake water [14].

## Conclusions

PAM is a rare but devastating central nervous system infection that can closely mimic acute bacterial meningitis, often leading to diagnostic delays. This case highlights the importance of considering PAM in patients presenting with rapidly progressive meningoencephalitis and compatible epidemiological exposure, particularly recent nasal exposure to lake water.

Despite the absence of definitive parasitological confirmation, the combination of clinical presentation, CSF findings, and characteristic neuroimaging features supported a presumptive diagnosis. The patient’s favorable clinical course suggests that early empirical therapy may improve outcome when clinical suspicion is high. Prompt recognition and timely treatment remain essential in this otherwise frequently fatal condition.

## References

[1] Visvesvara GS (2010). Amebic meningoencephalitides and keratitis: challenges in diagnosis and treatment. Curr Opin Infect Dis.

[2] Linam WM, Ahmed M, Cope JR (2015). Successful treatment of an adolescent with Naegleria fowleri primary amebic meningoencephalitis. Pediatrics.

[3] Capewell LG, Harris AM, Yoder JS (2015). Diagnosis, clinical course, and treatment of primary amoebic meningoencephalitis in the United States, 1937-2013. J Pediatric Infect Dis Soc.

[4] Yoder JS, Eddy BA, Visvesvara GS, Capewell L, Beach MJ (2010). The epidemiology of primary amoebic meningoencephalitis in the USA, 1962-2008. Epidemiol Infect.

[5] Cope JR, Ali IK (2016). Primary amebic meningoencephalitis: what have we learned in the last 5 years?. Curr Infect Dis Rep.

[6] Gharpure R, Bliton J, Goodman A, Ali IK, Yoder J, Cope JR (2021). Epidemiology and clinical characteristics of primary amebic meningoencephalitis caused by Naegleria fowleri: a global review. Clin Infect Dis.

[7] Visvesvara GS (2015). Pathogenic and opportunistic free-living amebae. Manual of Clinical Microbiology, 11th Edition.

[8] Martinez AJ, Visvesvara GS (1997). Free-living, amphizoic and opportunistic amebas. Brain Pathol.

[9] Siddiqui R, Khan NA (2014). Primary amoebic meningoencephalitis caused by Naegleria fowleri: an old enemy presenting new challenges. PLoS Negl Trop Dis.

[10] Singh P, Kochhar R, Vashishta RK, Khandelwal N, Prabhakar S, Mohindra S, Singhi P (2006). Amebic meningoencephalitis: spectrum of imaging findings. AJNR Am J Neuroradiol.

[11] Qvarnstrom Y, Visvesvara GS, Sriram R, da Silva AJ (2006). Multiplex real-time PCR assay for simultaneous detection of Acanthamoeba spp., Balamuthia mandrillaris, and Naegleria fowleri. J Clin Microbiol.

[12] Cope JR, Conrad DA, Cohen N, Cotilla M, DaSilva A, Jackson J, Visvesvara GS (2016). Use of the novel therapeutic agent miltefosine for the treatment of primary amebic meningoencephalitis: report of 1 fatal and 1 surviving case. Clin Infect Dis.

[13] Vargas-Zepeda J, Gómez-Alcalá AV, Vásquez-Morales JA, Licea-Amaya L, De Jonckheere JF, Lares-Villa F (2005). Successful treatment of Naegleria fowleri meningoencephalitis by using intravenous amphotericin B, fluconazole and rifampicin. Arch Med Res.

[14] McLaughlin A, O'Gorman T (2019). A local case of fulminant primary amoebic meningoencephalitis due to Naegleria fowleri. Rural Remote Health.

